# Proinflammatory cytokines suppress stemness-related properties and expression of tight junction in canine intestinal organoids

**DOI:** 10.1007/s11626-024-00936-w

**Published:** 2024-06-24

**Authors:** Meg Nakazawa, Itsuma Nagao, Yoko M. Ambrosini

**Affiliations:** 1 Department of Veterinary Clinical Sciences, College of Veterinary Medicine, Washington State University, Pullman, Washington, USA; 2 Department of Veterinary Internal Medicine, Graduate School of Agricultural and Life Sciences, The University of Tokyo, Tokyo, Japan

**Keywords:** Canine models, Organoids, Proinflammatory cytokines, Stem cell markers, Membrane integrity

## Abstract

Recent advancements in canine intestinal organoid research have paved the way for the development of enhanced in vitro models, crucial for exploring intestinal physiology and diseases. Despite these strides, there is a notable gap in creating specific in vitro models that focus on intestinal inflammation. Our study aims to bridge this gap by investigating the impact of proinflammatory cytokines on canine intestinal epithelial cells (IECs) within the context of organoid models. Canine intestinal organoids were treated with proinflammatory cytokines TNF-α, IFN-γ, and IL-1β. The expression of stem cell markers *Lgr5*, *Sox9*, *Hopx*, and *Olfm4* was evaluated through RT-qPCR, while membrane integrity was assessed using immunofluorescence staining for tight junction proteins and transport assays for permeability. IFN-γ significantly decreased *Lgr5* expression, a key intestinal stem cell marker, at both 24 and 48 h post-treatment (*p*=0.030 and *p*=0.002, respectively). Conversely, TNF-α increased *Olfm4* expression during the same intervals (*p*=0.018 and *p*=0.011, respectively). A reduction in EdU-positive cells, indicative of decreased cell proliferation, was observed following IFN-γ treatment. Additionally, a decrease in tight junction proteins E-cadherin and ZO-1 (*p*<0.001 and *p*=0.003, respectively) and increased permeability in IECs (*p*=0.012) were noted, particularly following treatment with IFN-γ. The study highlights the profound impact of proinflammatory cytokines on canine IECs, influencing both stem cell dynamics and membrane integrity. These insights shed light on the intricate cellular processes underlying inflammation in the gut and open avenues for more in-depth research into the long-term effects of inflammation on intestinal health.

**Supplementary Information:**

The online version contains supplementary material available at 10.1007/s11626-024-00936-w.

## Introduction

The investigation of intestinal epithelial cells (IECs) is critical due to their constant exposure to various external stresses such as dietary substances (Yu 2012), environmental chemicals (Lerner 2007), and enterobacteria (Okumura and Takeda 2017). This exposure often triggers excessive inflammation in IECs, causing cellular damage and compromising intestinal function (Levine and Wine 2013). Such inflammation is a key feature of inflammatory bowel diseases (IBD) in humans, including ulcerative colitis and Crohn’s disease (CD) (Trakman *et al*. 2022). Interestingly, dogs also naturally develop IBD-like conditions, positioning them as valuable models for human IBD research (Kol *et al*. 2016; Vázquez-Baeza *et al*. 2016). Both species suffer significant health impacts from IBD, yet a thorough understanding of its pathophysiology and effective treatments is hindered by the limitations of current in vitro models (Joshi *et al*. 2022).

In canine IBD, cytokines such as tumor necrosis factor-α (TNF-α), interferon-γ (IFN-γ), and interleukin-1β (IL-1β) are key players in disease pathogenesis. IFN-γ, secreted by T lymphocytes and specialized immune cells, has been shown to be elevated in the gut of dogs with IBD (German *et al*. 2000). Similarly, IL-1β, which are predominantly produced by innate immune cells, levels are elevated in the gut during canine IBD (Konstantinidis *et al*. 2021). TNF-α, predominantly produced by activated monocytes and macrophages, is also significantly increased in the gut of dogs with IBD (Kołodziejska-Sawerska *et al*. 2013). This elevation in cytokine levels underscores their integral role in the inflammatory processes of canine IBD.

Recent research further reveals that these proinflammatory cytokines’ role in intestinal inflammation extends beyond its direct immune activation. There has been controversial findings in studies trying to understand the effects of proinflammatory cytokines on intestinal cells, especially during heightened inflammation in IECs.

Treatment with proinflammatory cytokines has shown variable effects on stem cell populations, from enhancement (Bradford *et al*. 2017) to reduction (Saito *et al*. 2021), while also known to impair tight junction protein expression (Utech *et al*. 2005; Al-Sadi *et al*. 2016; Kaminsky *et al*. 2021). This disruption leads to increased penetration of luminal antigens into the intestinal lining, thereby intensifying the inflammatory response. This multi-faceted impact of proinflammatory cytokines underscores the complex nature of cytokine involvement in canine IBD and highlights the importance of understanding these mechanisms for developing effective treatments. However, these studies predominantly utilized enterocyte monolayers from cancer-derived cell lines (Van De Walle *et al*. 2010), which inadequately represent the full spectrum of intestinal cell functions, particularly regarding proliferation and differentiation. This limitation impedes a comprehensive understanding of IEC behavior. Furthermore, the lack of in vitro models for studying inflammatory cytokine impacts on canine IECs remains a significant gap.

The introduction of three-dimensional (3D) cultures of intestinal epithelial cells, termed intestinal organoids, has revolutionized intestinal biology research (Sato *et al*. 2009). Originating from primary intestinal stem cells, these organoids accurately mimic the intestinal structure and comprise various cell types of the intestinal lining, including enterocytes, goblet cells, Paneth cells, and enteroendocrine cells (Kawasaki *et al*. 2022). Notably, the implementation of organoid technology in the study of canine IECs has been a significant stride (Kramer *et al*. 2020). These canine intestinal organoids have been shown to effectively represent the physiological characteristics of these cells (Chandra *et al*. 2019; Ambrosini *et al*. 2020). This development not only propels the study of canine gut biology and associated disease mechanisms but also enriches our comprehension of intestinal health and disorders across both veterinary and human medical fields. The potential of these organoids in providing insights into various aspects of gut biology is immense, bridging critical gaps in our understanding and offering pathways for novel therapeutic interventions.

Our research aims to fill the knowledge gaps in understanding canine gut physiology and its response to proinflammatory cytokines. We are examining how canine IECs react to different cytokines and evaluating these responses using quantitative real-time RT-PCR (RT-qPCR) analysis, immunofluorescence staining, and transport assays. This study seeks to deepen our insight into the complexities of canine gut health and its reaction to inflammatory stimuli.

## Materials and methods

### Donor recruitment and intestinal sample collection

Colonic biopsy tissue samples were obtained from three clinically healthy canines during dental procedures at the Washington State University Veterinary Teaching Hospital Community Service. Detailed information about the donor dogs is provided in Supplementary Table 1. These dogs, aged between 1 and 12 yr, underwent thorough physical examinations and blood tests, and had no history of chronic diseases affecting major organs such as the heart, liver, kidneys, or intestines. Inclusion in the study was limited to dogs that were considered suitable for elective anesthesia. The research was approved and carried out with the endorsement of the Washington State University IACUC, under ASAF#6993.

### Generation and maintenance of canine colonoids

The isolation of colonic crypts from biopsy samples was conducted using a method modified from a previous report (Chandra *et al*. 2019). In brief, colonic biopsy pieces were washed five times in ice-cold Dulbecco’s phosphate-buffered saline (PBS, Thermo Fisher Scientific, Waltham, MA) with 1× penicillin/streptomycin (Thermo Fisher Scientific). These biopsies were then finely chopped with scissors and incubated in a rotating 15-mL conical tube containing a 30 mM EDTA solution (Thermo Fisher Scientific) at 4°C for 30 min. After incubation, the tubes were allowed to settle, and the supernatant, containing the crypts, was collected. These crypts were then centrifuged at 200 × *g* at 4°C for 5 min to form a pellet. This pellet was resuspended in Matrigel (Corning, Glendale, AZ) and placed into 48-well culture plates (Thermo Fisher Scientific) at 30 μL per well. After solidifying the Matrigel domes in a 37°C incubator for 10 min, 300 μl of organoid expansion medium (EM) was added to each well. The EM composition was based on a previous study (Chandra *et al*. 2019) and included DMEM/F12 (Thermo Fisher Scientific) supplemented with 2 mM GultaMAX (Thermo Fisher Scientific), 10 mM HEPES (Thermo Fisher Scientific), 1× penicillin/streptomycin (Thermo Fisher Scientific), 10% (vol/vol) conditioned medium of Noggin (Heijmans *et al*. 2013), 20% (vol/vol) conditioned medium of R-spondin, 100 ng/mL recombinant murine Wnt-3a (PeproTech, Cranbury, NJ), 50 ng/mL murine epidermal growth factor (EGF) (PeproTech), 10 nM Gastrin (Sigma-Aldrich, St. Louis, MO), 500 nM A-83-01 (Sigma-Aldrich), 10 μM SB202190 (Sigma-Aldrich), 1 mM N-acetyl-l-Cysteine (MP Biomedicals, Irvine, CA), 10 mM nicotinamide (Sigma-Aldrich), 1× B27 supplement (Thermo Fisher Scientific), 1× N2 MAX media supplement (R&D Systems, Minneapolis, MN), and 100 μg/mL Pimocin (Thermo Fisher Scientific). The medium was refreshed every other day. For the first 2 days post-crypt isolation, 10 μM Y-27632 (Stem Cell Technologies, Vancouver, Canada) and 2.5 μM CHIR 99021 (Stem Cell Technologies) were included in the medium. The colonoids were passaged every 6–8 d. To dissolve the Matrigel domes, we used Cell Recovery Solution (Corning) at 4°C for 30 min. The colonoids were then separated using TrypLE Express (Thermo Fisher Scientific), centrifuged, and resuspended in Matrigel for re-plating in a 48-well plate at a 1:6-8 dilution.

### Cytokine treatment of canine colonoids

**Figure 1.**
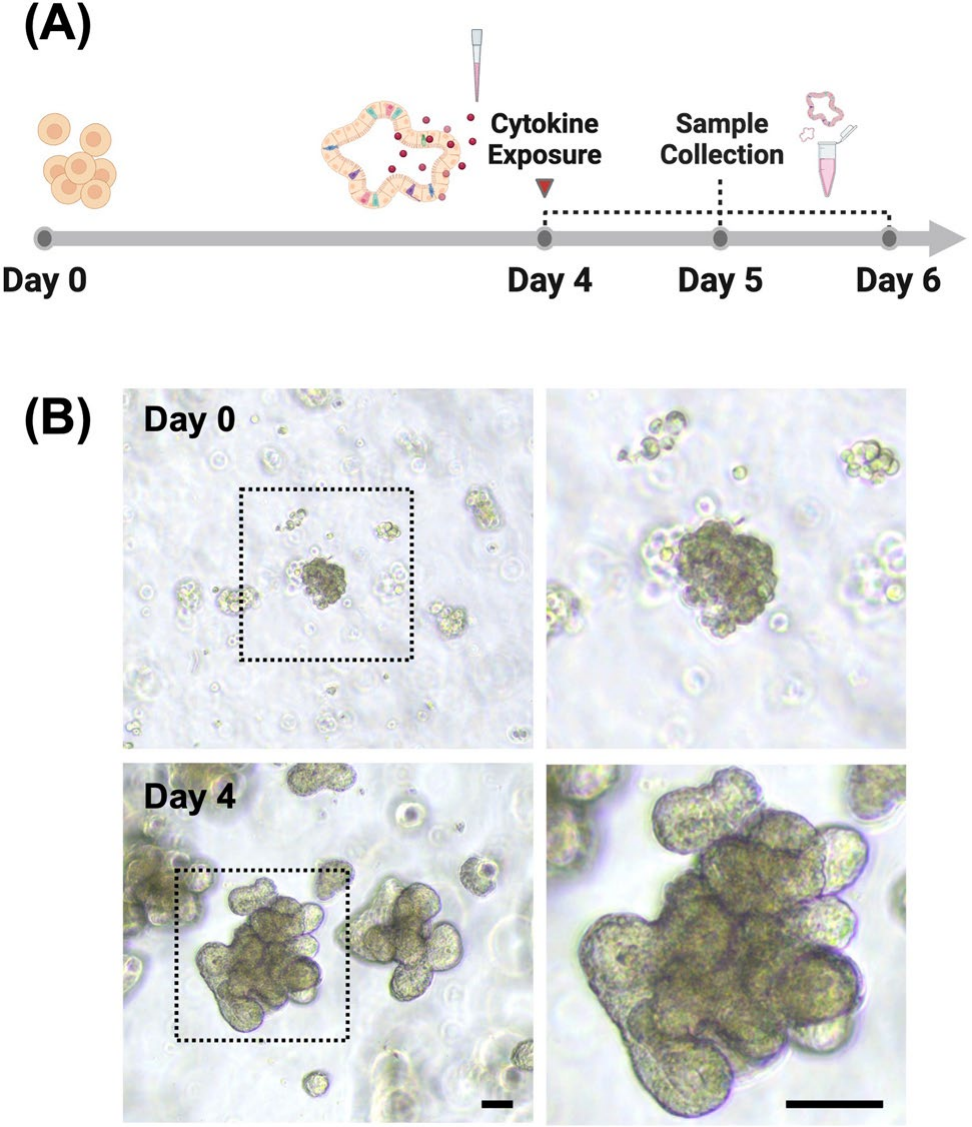
In vitro simulation of intestinal inflammation using canine colonoids. (*A*) A schematic representation of the flow of the inflammatory cytokine treatment. The canine colonoids were initially cultured for 4 d, followed by treating with inflammatory cytokines (30 ng/mL of TNF-α, IFN-γ, IL-1β, respectively). The colonoids were then collected at 0 h, 24 h and 48 h after cytokine treatment for further analysis. This schematic was created with BioRender.com. (*B*) Representative phase-contrast microscopy images displaying canine colonoids at day 0 and day 4. *Scale bar* = 50 μm.

A schematic image of flow of cytokine treatment is shown in Fig. 1*A*. Some variations in organoid sizes are present; however, a definite growth in size from day 0 to day 4 was noted, as evidenced by the measurable increase in their dimensions (Fig. 1*B*). Four days post-passage, the canine colonoids were subjected to treatment with 30 ng/mL of recombinant canine cytokines, i.e., TNF-α, IFN-γ, or IL-1β (R&D Systems), each added to the EM. The concentration of these proinflammatory cytokines was set based on a previous study (Onozato *et al*. 2020; Lee *et al*. 2021; Saito *et al*. 2021).

### Quantitative real-time RT-PCR

Total RNA was isolated from the colonoids 0-, 24-, and 48-h post-cytokine treatment utilizing the RNeasy Mini Kit (Qiagen, Venlo, Netherlands). This was followed by the synthesis of complementary DNA using the High-Capacity cDNA Reverse Transcription Kit (Thermo Fisher Scientific). The gene expression levels of stem cell markers—leucine-rich repeat-containing G-protein coupled receptor 5 (*Lgr5*), SRY-box transcription factor 9 (*Sox9*), olfactomedin 4 (*Olfm4*), homeodomain only protein (*Hopx*), and axis inhibition protein 2 (*Axin2*) (Smolders *et al*. 2013; Bongiovanni *et al*. 2020; Sahoo *et al*. 2023)—and apoptosis markers—caspase 3 (*Casp3*) and caspase 8 (*Casp8*) (Del Puerto *et al*. 2010; Wilke *et al*. 2012)—were quantified through PowerUp SYBR Green Master Mix (Thermo Fisher Scientific) on a CFX96 Touch Real-Time PCR Detection System (Bio-Rad, Hercules, CA). RT-qPCR reactions, performed in triplicate, allowed for the calculation of relative gene expression using the ΔΔCt method. Internal controls for these reactions included *HMBS*, *HPRT1*, and *SDHA*, as previously reported (Peters *et al*. 2007). The results were then normalized against the average relative gene expression levels observed in the control groups. Details of all primers used are provided in Supplementary Table 2.

### Immunocytochemistry

Colonoids were fixed using 4% paraformaldehyde (Thermo Fisher Scientific) for 15 min at room temperature. This was followed by permeabilization with 0.3% Triton X-100 (Thermo Fisher Scientific) for 15 min and blocking with 2% bovine serum albumin (BSA) (Cytiva, Marlborough, MA) for an hour. The colonoids were then incubated with primary antibodies: E-cadherin (36/E-cadherin, 1:200, BD Biosciences, Franklin Lakes, NJ) and Zonula occludens-1 (ZO-1) polyclonal antibody (61-7300, 1:50, Thermo Fisher Scientific). Afterward, they were treated with Alexa Fluor 555-conjugated Goat Anti-Rabbit IgG H&L secondary antibody (1:1000, Abcam, Cambridge, UK) for 1 h at room temperature, post-PBS wash. Nuclei was stained using DAPI (1:1000, Thermo Fisher Scientific). Following another PBS wash, the colonoids were suspended in Prolong Gold Antifade reagent (Thermo Fisher Scientific) and mounted on a glass bottom dish (Matsunami, Osaka, Japan).

Fluorescence imaging was conducted using a white light point scanning confocal microscope (SP8-X, Leica, Wetzlar, Germany), and image processing was done using LAS X software (Leica). For quantitative analysis of E-cadherin and ZO-1 expression, we randomly selected ten to fifteen random fields of view from two biological replicates. ImageJ software was used for fluorescence image analysis (Schneider *et al*. 2012). For E-cadherin, line plot analysis was conducted at three random positions per colonoid. ZO-1 expression was assessed by measuring the mean area intensity of the apical area of colonoids using a line width of 60.

### 5-Ethynyl-2-deoxyuridine assay

In the 5-ethynyl-2-deoxyuridine (EdU) assay, colonoids treated with cytokines for 24 and 48 h were incubated in EM supplemented with 10 μM of EdU stock solution for 3 h. Post-incubation, staining was carried out using Click-iT EdU Imaging Kits (Thermo Fisher Scientific), adhering to the guidelines provided by the manufacturer. Subsequently, the nuclei were stained with DAPI. The proportion of EdU-positive cells was determined by comparing the count of EdU-positive cells to the total number of nuclei. Fluorescence imaging was conducted using a white light point scanning confocal microscope (SP8-X, Leica), and image processing was completed with LAS X software (Leica). For analysis, 15 separate fields of view were randomly chosen from two biological replicates.

### Permeability assay

The fluorescein isothiocyanate (FITC)-dextran permeability assay was conducted 48 h post-cytokine treatment, as adapted from a previous study (Crawford *et al*. 2022). For this, colonoids were first cultivated on glass bottom dishes (Matsunami) for 4 d following passage. These colonoids were then exposed to 30 ng/mL of various inflammatory cytokines (TNF-α, IFN-γ, or IL-1β) for 48 h. Subsequently, the colonoids were incubated with EM infused with 5 μg/mL of 4 kDa FITC-dextran (Sigma-Aldrich) for 1 h. After this, the Matrigel domes were rinsed three times with PBS, and the colonoids were visualized using the EVOS FL Imaging System (Thermo Fisher Scientific). For analysis, twenty colonoids were randomly chosen. The intensity of FITC-dextran within each colonoid was measured at three different points and then normalized against the average FITC-dextran intensity outside the colonoid, using ImageJ software (Schneider *et al*. 2012).

### Statistical analyses

Quantitative data in our study were processed using R v 3.4.1 (The R Foundation, Indianapolis, IN), and visualized using GraphPad Prism version 10.1.1 (GraphPad Software, Boston, MA). We employed the Kruskal-Wallis test followed by a Dunn’s multiple comparison test for RT-qPCR to compare differences among the control group, and groups treated with TNF-α, IFN-γ, and IL-1β. Kruskal-Wallis test followed by a Bonferroni post hoc test was applied to immunocytochemistry, the proportion of EdU-positive cells, and the FITC-dextran permeability ratio. Results are reported as mean ± standard error of the mean (SEM), and a *p*-value of 0.05 or less was considered to denote statistical significance. In all our tests, we adhered to the threshold of *p*<0.05 to define statistical significance.

## Results

### Inflammatory cytokines influence the expression of major ISC markers

**Figure 2.**
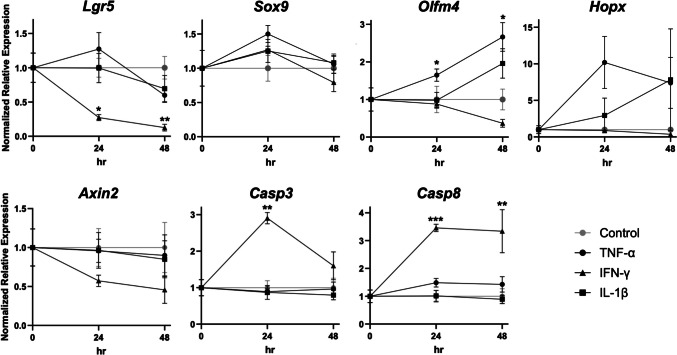
Expression profiles of stemness-associated genes (*Lgr5*, *Sox9*, *Olfm4*, *Hopx*, and *Axin2*) and apoptosis-related gene (*Casp3* and *Casp8*) in canine colonoids following proinflammatory cytokine treatments. Gene expressions were assessed using quantitative reverse transcription polymerase chain reaction (RT-qPCR). In this *graph*, each value of cytokine-treated group (TNF-α, IFN-γ, or IL-1β) was normalized using the average of control at 0, 24, and 48 h after treatment. The *error bars* represent the standard error of the mean (SEM). *p<0.05, **p<0.01, ***p<0.001.

We evaluated the mRNA expression levels of stemness-related genes using quantitative RT-PCR. Post-cytokine treatment, a time-dependent decrease in the expression of the stem cell marker *Lgr5* was noted in groups treated with TNF-α, IFN-γ, and IL-1β, contrasting with a slight increase in the control group, as shown in Fig. 2. Significantly, there was a marked reduction in *Lgr5* expression at both 24 (*p*=0.030) and 48 h (*p=*0.002) following IFN-γ treatment when compared to the non-stimulated control group.

In terms of other stem cell markers, *Sox9* did not exhibit significant changes at either of the two time points in comparison to the control group. Notably, TNF-α treatment led to a significant upregulation of *Olfm4* expression at 24 (*p*=0.018) and 48 h (*p*=0.011) post-treatment. The expression of *Hopx*, a marker of quiescent stem cells, showed a slight increase following treatment with IFN-γ and IL-1β, but these changes did not reach statistical significance. Our results indicated a noticeable reduction in *Axin2* expression at both 24 and 48 h in the IFN-γ group, though these changes did not reach statistical significance.

To further explore whether inflammatory cytokines generally inhibit epithelial cell proliferation and potentially induce apoptotic cell death, we performed qPCR analyses for *Casp3* and *Casp8*. Our results demonstrated significant expression of *Casp3* at 24 h (*p*=0.006) in the IFN-γ group, and significant expression of *Casp8* at both 24 and 48 h (*p*<0.001 and *p*=0.006, respectively) in the same group.

### IFN-γ and IL-1β suppresses the cell proliferation

**Figure 3.**
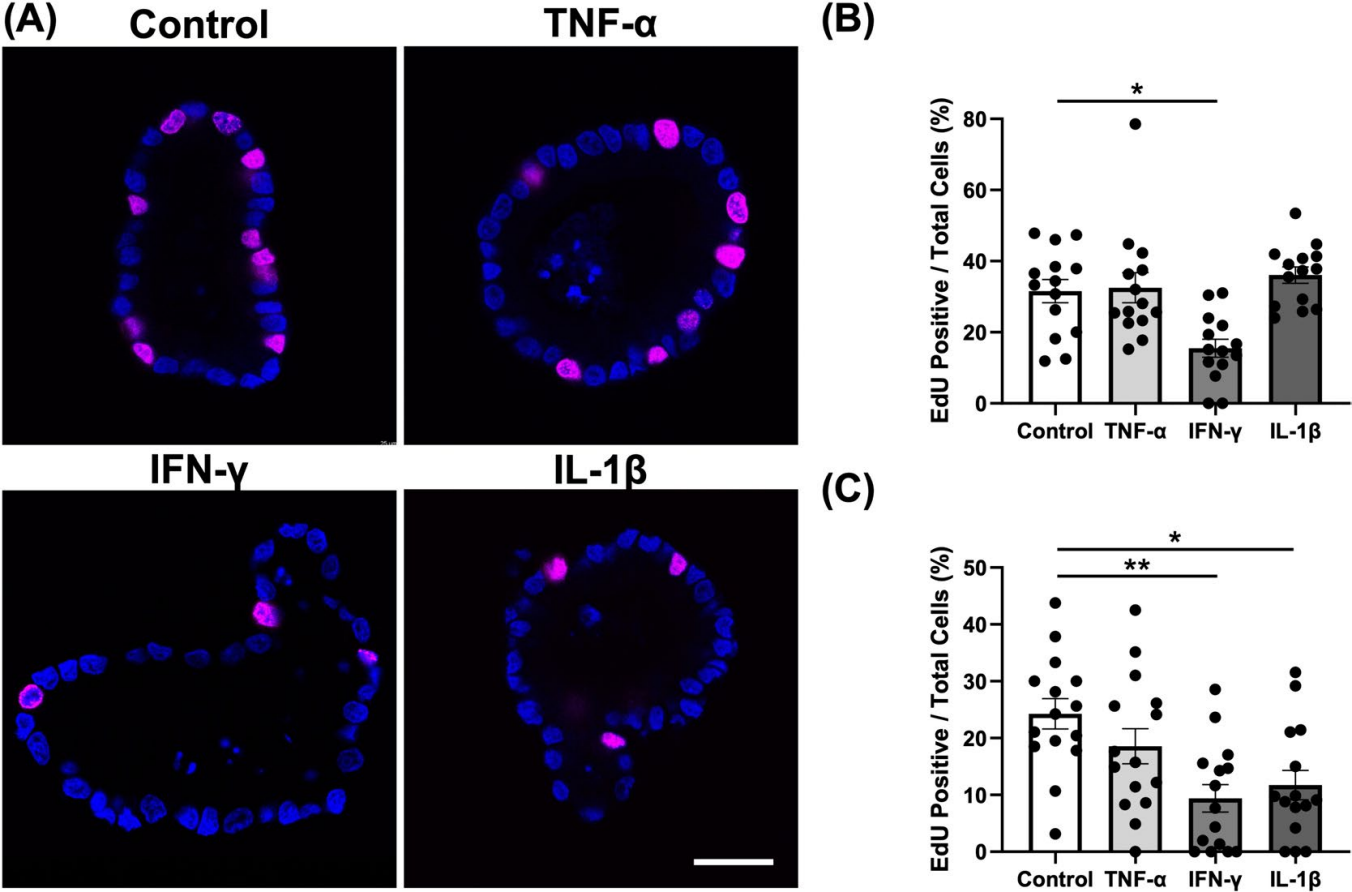
Impact of proinflammatory cytokine exposure on cell proliferation dynamics. (*A*) Representative confocal images of colonoids treated with EdU assay at 48 h after cytokine treatment (TNF-α, IFN-γ, or IL-1β). Active proliferative cells (EdU, cyan) and nuclei (DAPI, blue) were stained. Scale bar = 50 μm. (*B*) Proportion of EdU positive cells against total cells at 24 h and (*C*) 48 h after cytokine treatment. *p<0.05, **p<0.001.

To evaluate cell proliferation following exposure to cytokines, we quantified the ratio of EdU-positive cells (Fig. 3*A*). After 24 h, treatment with IFN-γ resulted in a notable decrease in the percentage of EdU-positive cells (*p*=0.012), as illustrated in Fig. 3*B*. Furthermore, at the 48-h mark, both IFN-γ and IL-1β treatments led to a reduction in the ratio of EdU-positive cells in comparison to the control group (*p=*0.003, *p*=0.031, respectively) (Fig. 3*C*).

### TNF-α, IFN-γ, and IL-1β reduce the tight junction protein expression

**Figure 4.**
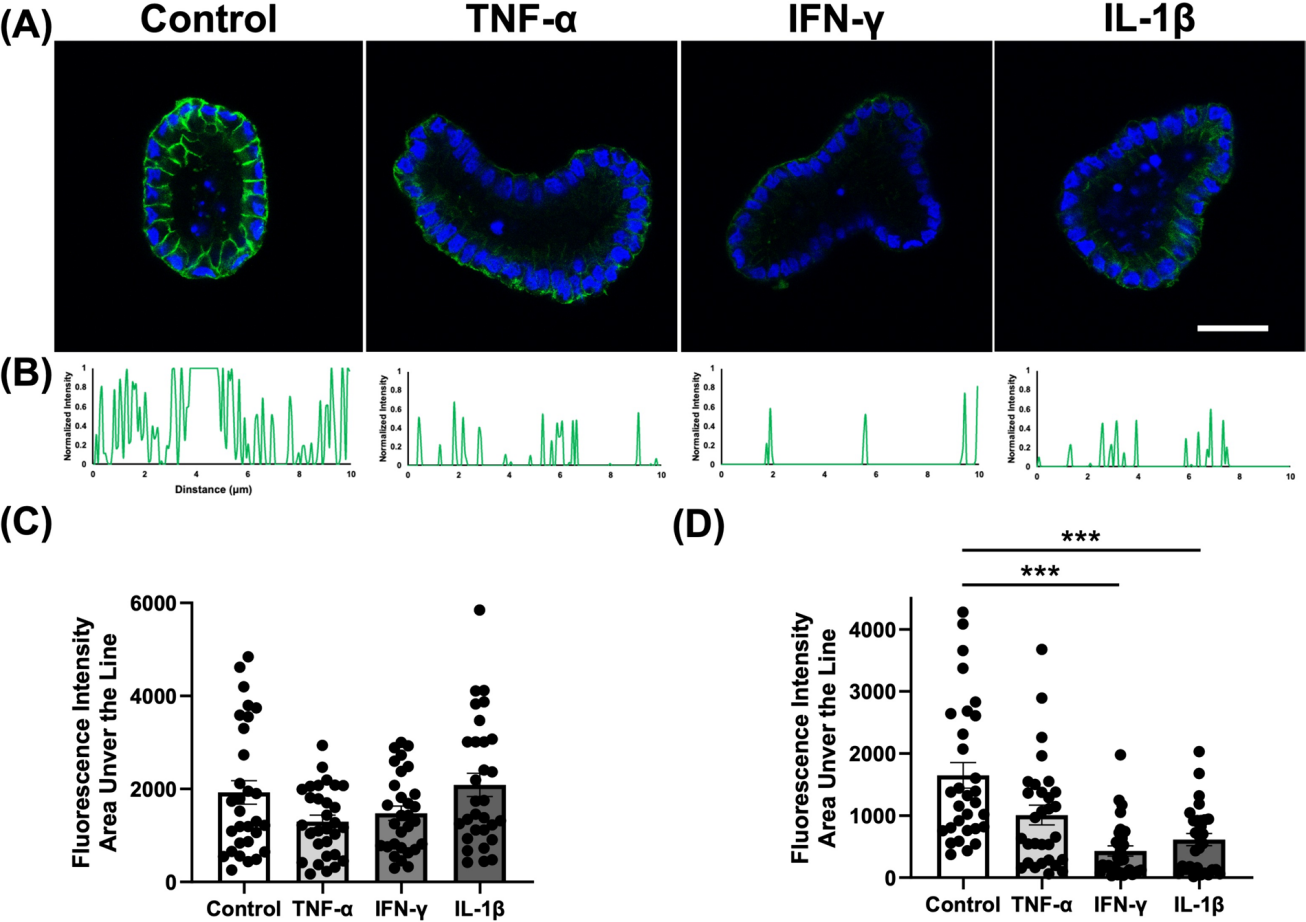
Influence of proinflammatory cytokines on E-cadherin expression. (*A*) Immunofluorescence staining of E-cadherin (*green*) and nuclei (*blue*) at 48 h after treating colonoids with cytokines (TNF-α, IFN-γ, or IL-1β). *Scale bar* = 50 μm. (*B*) Line plot scan at an arbitrary position of each immunofluorescence image of E-cadherin (distance 10 μm). (*C*) Comparison of area under the line obtained from the line plot scan of colonoids at 24 h and (*D*) 48 h after cytokine treatment. ***p<0.001.

**Figure 5.**
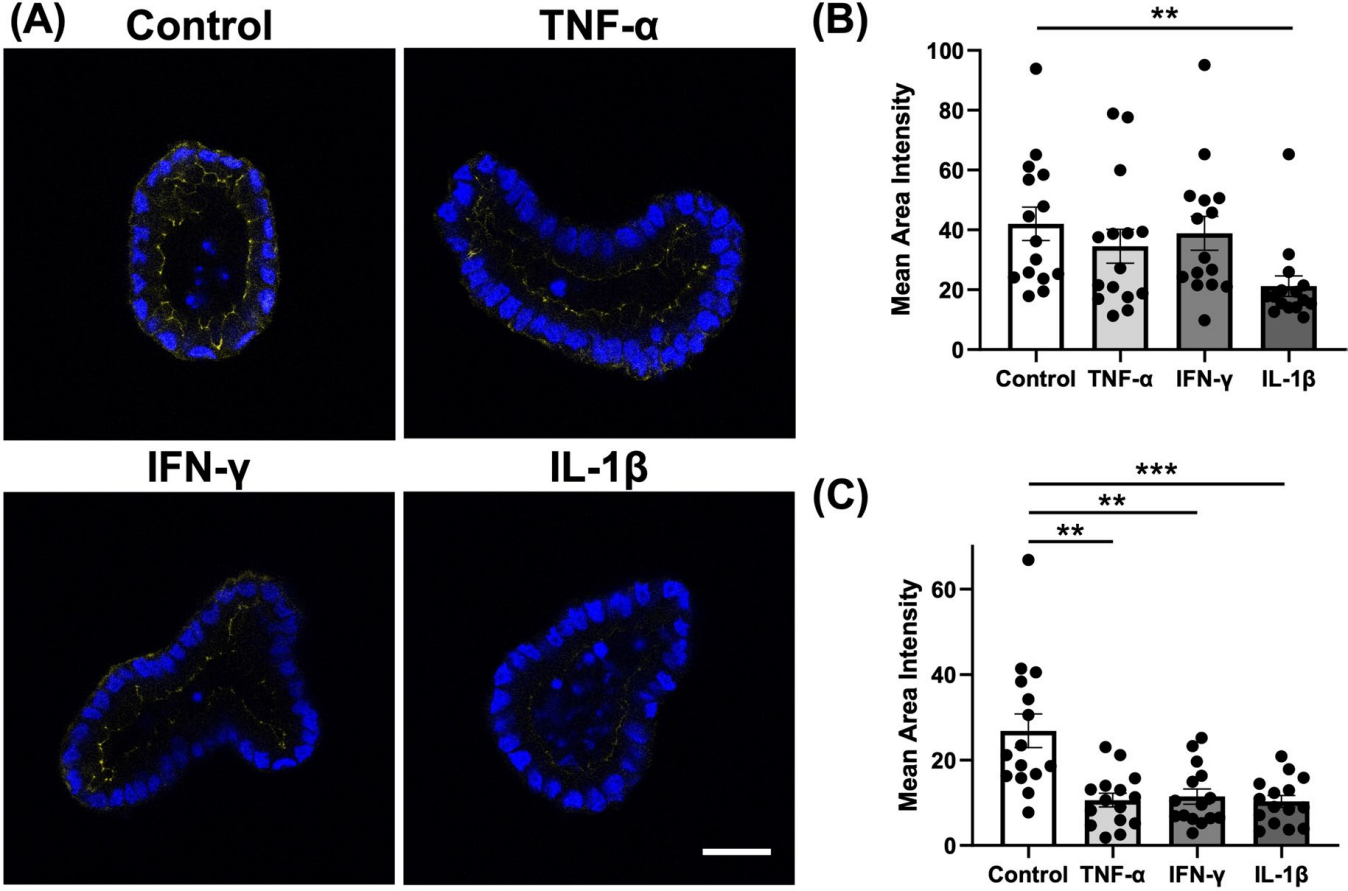
Influence of proinflammatory cytokines on ZO-1 expression. (*A*) Immunofluorescence staining of ZO-1 (*yellow*) and nuclei (*blue*) at 48 h after treating colonoids with cytokines (TNF-α, IFN-γ, or IL-1β). *Scale bar* = 50 μm. (*B*) Comparison of mean area intensity of apical area of colonoids at 24 h and (*C*) 48 h after cytokine treatment. **p<0.01, ***p<0.001.

Immunofluorescence staining of E-cadherin, a tight junction protein, was evaluated at 24 and 48 h post-cytokine treatment (Fig. 4). Line plot analysis, as shown in Fig. 4*C*, *D*, indicated that TNF-α, IFN-γ, and IL-1β treatments led to a time-dependent reduction in E-cadherin expression. Notably, a significant decrease in E-cadherin levels compared to the control group was evident only 48 h after treatment with IFN-γ and IL-1β (*p*<0.001). Subsequently, we examined the expression of another tight junction protein, ZO-1 (Fig. 5). IL-1β treatment resulted in a significant reduction in ZO-1’s mean area intensity as early as 24 h post-treatment (*p*=0.003), detailed in Fig. 5*B*. Furthermore, at the 48-h mark, all three inflammatory cytokines—TNF-α, IFN-γ, and IL-1β—showed a time-dependent decrease in the mean area intensity of ZO-1 (*p*=0.001, *p*=0.003, and *p*<0.001, respectively) (Fig. 5*C*).

### TNF-α and IFN-γ treatment increased epithelial permeability

**Figure 6.**
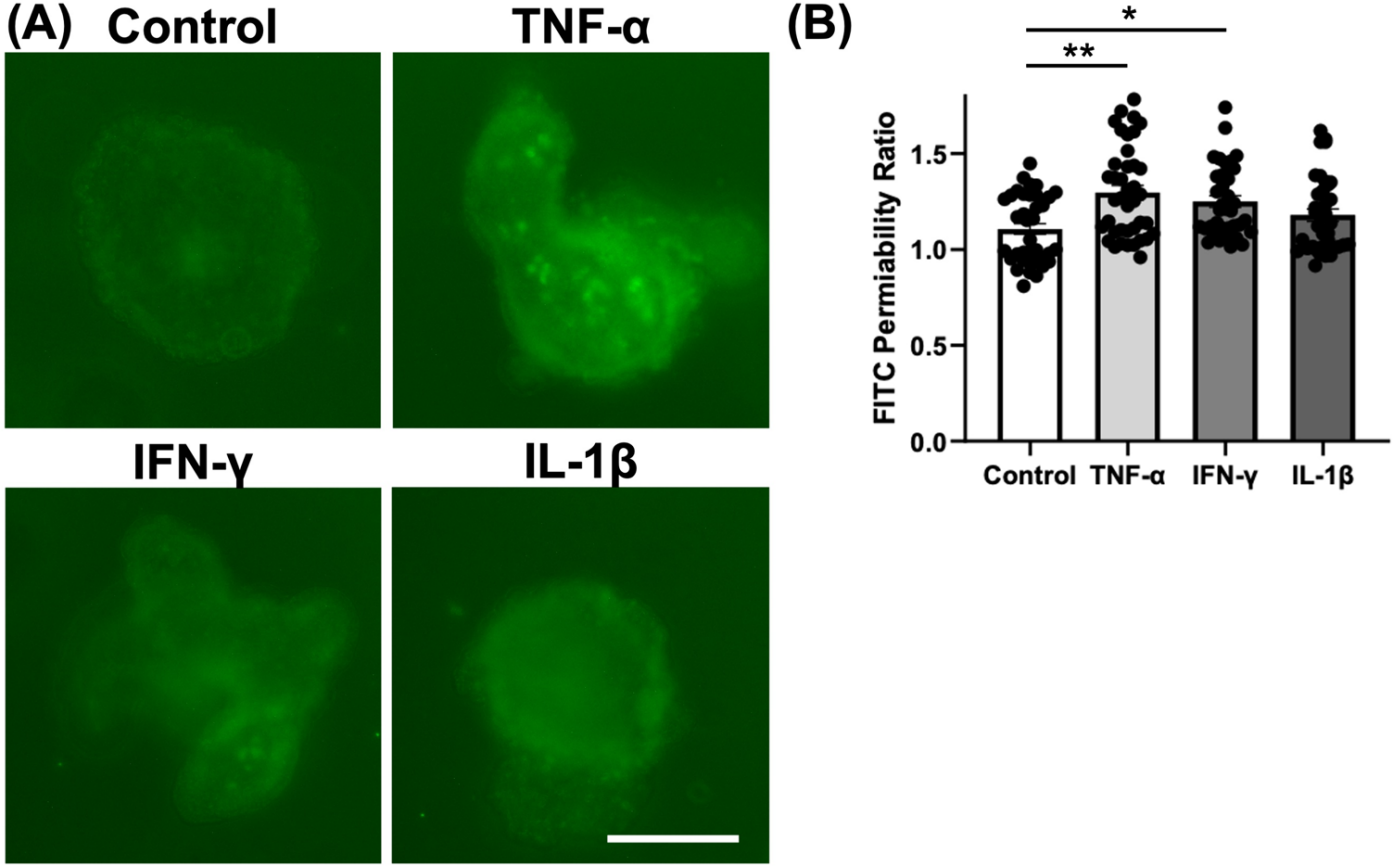
Compromise in intestinal barrier function after exposure to proinflammatory cytokines. (*A*) Representative fluorescence microscope image of colonoids treated with inflammatory cytokines (TNF-α, IFN-γ, or IL-1β) for 48 h, followed by treating with 5 μg/mL of 4 kDa fluorescein isothiocyanate (FITC)-dextran for 1 h. *Scale bar* = 100 μm. (*B*) The proportion of FITC intensity inside against outside of colonoids at 48 h after cytokine treatment was calculated. The data from two biological replicates and 20 technical replicates were used for analysis. **p<0.01, ***p<0.001.

Forty-eight hours following cytokine treatment, we evaluated the tight junction barrier function using a FITC-dextran permeability assay (Fig. 6*A*). Analysis of the FITC permeability ratio indicated that both TNF-α and IFN-γ treatments significantly elevated the permeability ratio after a 48-h period (*p*=0.002 and *p*=0.012, respectively) (Fig. 6*B*).

## Discussion

This study revealed significant changes in the expression of crucial gut stem cell markers following exposure to proinflammatory cytokines. Specifically, there was a notable decrease in *Lgr5* expression after IFN-γ treatment, while TNF-α exposure led to an increase in *Olfm4* expression. Additionally, proteins critical to maintaining tight junction integrity exhibited a marked decrease, particularly after IFN-γ exposure. Transport assay results corroborated these findings, indicating a compromise in membrane integrity due to proinflammatory cytokine exposure. These outcomes highlight the varied responses of IECs to different proinflammatory cytokines, underscoring the complexity of their roles in gut physiology.

In our study, we observed that IFN-γ significantly decreased the expression of *Lgr5* at both 24 and 48 h after treatment. In contrast, TNF-α was found to increase the expression of *Olfm4* during these same intervals. Interestingly, although not statistically significant, *Olfm4* expression showed a decrease following 48 h of IFN-γ treatment. *Lgr5* is a crucial marker for intestinal stem cells, playing a key role in the Wnt signaling pathway which is vital for the differentiation of these cells (Haegebarth and Clevers 2009). On the other hand, *Olfm4* is another intestinal stem cell marker, known for its strict expression in the intestinal crypt and its higher expression levels compared to *Lgr5*, making it a more robust marker for these cells (Schuijers *et al*. 2014). *Olfm4*, a secreted protein, is involved in various cell signaling pathways that are key to cell proliferation, regeneration, and apoptosis (Liu and Rodgers 2016). The observed increase in *Olfm4*-positive stem cells following treatment with TNF-α could be indicative of stem cell recovery and regeneration processes in response to inflammatory damage within the gut (Bradford *et al*. 2017). This suggests that *Olfm4* may not only be a marker of intestinal stem cells but also a participant in the cellular response to inflammation and tissue repair mechanisms (Kuno *et al*. 2021).

To investigate the effect of inflammatory cytokines on Wnt signaling, we measured β-catenin signaling components, including *Axin2*, which decreased at both 24 and 48 h following IFN-γ treatment, although these changes did not achieve statistical significance (Fig. 2). This observed trend of *Axin2* reduction aligns with findings from previous studies (Takashima *et al*. 2019). Our results suggest that IFN-γ impairs the expansion of LGR5+ stem cells while also disrupting β-catenin signaling.

Our qPCR studies revealed that IFN-γ’s impact on *Lgr5* expression was evident as early as 24 h post-treatment, with the effect intensifying at the 48-h mark. This trend corresponds with results from EdU incorporation studies, where we noted a substantial decrease in EdU-positive cells at 24 h following IFN-γ treatment. In contrast, organoids treated with TNF-α or IL-1β did not exhibit such a reduction. At 48 h, both IFN-γ- and IL-1β-treated organoids displayed diminished EdU-positive cells, whereas those treated with TNF-α did not show a similar decrease. These findings indicate a strong correlation between the proliferation capacity of IECs and *Lgr5* gene expression, reinforcing earlier research (Saito *et al*. 2021). This correlation underscores the importance of *Lgr5* in the regulation of IEC proliferation. While our study did not yield statistically significant results, we observed an increase in *Hopx* expression in cells treated with cytokines at both evaluated time points. Previous research has demonstrated that increased *Hopx* expression occurs following severe intestinal damage (Stewart *et al*. 2021). This suggests that a similar response might be present in canine IECs under similar conditions of stress or damage. Given the varying effects of proinflammatory cytokines on intestinal stem cell populations, further investigation is necessary. By delving deeper into these dynamics, we can gain more nuanced insights into how inflammation affects intestinal stem cells, paving the way for more targeted therapeutic approaches.

To determine whether inflammatory cytokines also inhibit epithelial cell proliferation and potentially induce apoptotic cell death in canine colonoids, we concurrently assessed the expression of *Casp3* and *Casp8*. Our analysis revealed significant expression of *Casp3* at 24 h in the IFN-γ-treated group, and significant expression of *Casp8* at both 24 and 48 h in the same group. These findings suggest that IFN-γ not only inhibits the expansion of LGR5+ stem cells but also activates apoptotic pathways in canine colonoids. The observed activation of apoptotic pathways following IFN-γ treatment is consistent with findings from previous studies (Takashima *et al*. 2019; Crawford *et al*. 2022). The increased expression of both *Casp3* and *Casp8* indicates a robust activation of apoptosis, which could contribute to the inflammation-associated tissue damage observed in these models.

In assessing membrane integrity, we observed a reduction in both ZO-1 and E-cadherin proteins, as determined through immunofluorescence staining. Line scan analysis highlighted a loss of distinct peaks, indicating diminished fluorescence signal presence. Complementing these results, a transport assay employing FITC-dextran permeability assay revealed an increase in permeability across IECs following cytokine treatment, particularly in intestinal organoids treated with TNF-α and IFN-γ, as previously reported (Crawford *et al*. 2022). The data suggest that proinflammatory cytokines reduce the expression of junctional proteins, leading to increased gut permeability, a condition often referred to as “leaky gut.” This heightened permeability may play a role in further recruiting local immune cells during intestinal inflammation, or it might contribute to compromised membrane integrity, a crucial defense mechanism against pathogenic invasion. The observed reduction in E-cadherin expression may suggest the induction of epithelial-mesenchymal transition (EMT) rather than merely changes in epithelial barrier function. Given that chronic inflammation can lead to intestinal neoplasia via EMT, this could be a valuable focus for future research.

One limitation of our study is the indirect method we employed to measure substrate accumulation within organoids during our transport assays. We utilized fluorescence intensity to assess intestinal membrane permeability, which, while informative, does not directly measure the quantity of substrate transported. The reliance on fluorescence imaging of FITC-dextran introduces uncertainties, particularly due to cellular autofluorescence, which can complicate the differentiation between the transportation of the fluorescent agent and inherent autofluorescence (Crawford *et al*. 2022; Ginga *et al*. 2022). Additionally, the penetration of fluorescence agents may be inefficient in the inner layers of the organoid, particularly when encapsulated by extracellular matrix (ECM) materials like Matrigel (Wolf 2008). This can create gradients in cell viability and function, further complicating the interpretation of our transport assays. Recently, we have developed an effective protocol for creating a two-dimensional (2D) monolayer derived from organoids. This monolayer forms a physiologically relevant intestinal epithelial interface, characterized by the expression of tight junctions. A key advantage of this system is the ability to measure transepithelial electrical resistance (TEER), which offers a functional analysis of cell junctions. This approach could enhance the accuracy and relevance of our studies, offering deeper insights into the cellular mechanisms at play in intestinal health and disease.

## Conclusion

Our study has successfully demonstrated the utility of canine intestinal organoids in evaluating the response of IECs to proinflammatory cytokines, thus contributing valuable insights into canine gut physiology. Upon exposure to proinflammatory cytokines, we observed a notable decrease in the expression of stem cell marker genes. Additionally, there was a significant reduction in the expression of tight junction proteins, corroborated by findings from transport assays that indicated a compromised membrane integrity following cytokine exposure. These findings reveal that short-term exposure to proinflammatory cytokines leads to diminished tight junction integrity in IECs and a reduction in the stem cell population. This information is crucial for understanding the initial cellular responses to inflammatory stimuli. Future research could extend these findings by exploring the effects of long-term exposure to proinflammatory cytokines, which will further deepen our understanding of the chronic impacts of inflammation on intestinal health.

## Supplementary information

ESM 1 Supplementary Table 1. Signalment of healthy dogs included this study. Signalment (breed, sex, and age) of healthy dogs used in this study is summarized. Supplementary Table 2. Primer information used in this study. Gene name, forward (F) and reverse (R) sequences and product size are listed. (DOCX 16439 kb)

## Data Availability

All data relevant to the study are included in the article or uploaded as supplementary information.
