# Supplementary material for: Proinflammatory cytokines suppress stemness-related properties and expression of tight junction in canine intestinal organoids
Source: In Vitro Cell Dev Biol Anim. 2024 Jun 24;60(8):916–25. doi: 10.1007/s11626-024-00936-w (PMC11419940; doi:10.1007/s11626-024-00936-w)
Supplement: Supplementary file 1 — Supplementary Table 1. Signalment of healthy dogs included this study. Signalment (breed, sex, and age) of healthy dogs used in this study is summarized. Supplementary Table 2. Primer information used in this study. Gene name, forward (F) and reverse (R) sequences and product size are listed. (DOCX 16439 kb) [file 11626_2024_936_MOESM1_ESM.docx]

**Appendix**

**Supplementary Table 1**. Signalment of healthy dogs included this study. Signalment (breed, sex, and age) of healthy dogs used in this study is summarized.

|  | | | |
| --- | --- | --- | --- |
| Case | Breed | Age | Sex |
| Dog1 | Australian Cattle Dog | 7y 6m | Castrated male |
| Dog2 | Boston Terrier | 11y 6m | Spayed female |
| Dog3 | Mixed Breed Dog | 1y 0m | Castrated male |
|  | | | |

**Supplementary Table 2**. Primer information used in this study. Gene name, forward (F) and reverse (R) sequences and product size are listed.


|  |  |  |  |  |
| --- | --- | --- | --- | --- |
| *Gene* |  | Sequences | Product length | Genbank accession number |
| *Lgr5* | F | GGCTCCACAGCCTAGAGACTTTAG | 109 | XM_038678819 |
|  | R | TTGTTGCTGTGAAATC CTAGTTCTTT |  |  |
| *Sox9* | F | TTCCGCGACGTGGACAT | 77 | NM_001002978 |
|  | R | TCGAATTCGTTGACGTCGAA |  |  |
| *Olfm4* | F | GTATCATGAATGTCAGCAAGC | 163 | XM_038569678 |
|  | R | CTGTAATATTCCAGAATTCTTCC |  |  |
| *Hopx* | F | GACCAGGTGGAGATTCTGG | 134 | XM_038556181 |
|  | R | GCCAGACGCTGCTTAAACC |  |  |
| *Axin2* | F | GGACAAATGCGTGGATACCT | 141 | XM_548025 |
|  | R | TGCTTGGAGACAATGCTGTT |  |  |
| *Casp3* | F | ATTATTCAGGCCTGCCGAGGTACA | 110 | NM_001419299 |
|  | R | TACAAGAAGTCCGCTTCGACTGGT |  |  |
| *Casp8* | F | ACAAGGGCATCATCTATGGCTCTGA | 70 | NM_001048029 |
|  | R | CCAGTGAAGTAAGAGGTCAGCTCAT |  |  |
| *SDHA* | F | GCCTTGGATCTCTTGATGGA | 92 | XM_535807 |
|  | R | TTCTTGGCTCTTATGCGATG |  |  |
| *HMBS* | F | TCACCATCGGAGCCATCT | 112 | XM_546491 |
|  | R | GTTCCCACCACGCTCTTCT |  |  |
| *HPRT1* | F | CACTGGGAAAACAATGCAGA | 123 | AY_283372 |
|  | R | ACAAAGTCAGGTTTATAGCCAACA |  |  |
|  |  |  |  |  |
